# Genome Sequencing and Comparative Analysis of *Stenotrophomonas acidaminiphila* Reveal Evolutionary Insights Into Sulfamethoxazole Resistance

**DOI:** 10.3389/fmicb.2018.01013

**Published:** 2018-05-17

**Authors:** Yao-Ting Huang, Jia-Min Chen, Bing-Ching Ho, Zong-Yen Wu, Rita C. Kuo, Po-Yu Liu

**Affiliations:** ^1^Department of Computer Science and Information Engineering, National Chung Cheng University, Chiayi, Taiwan; ^2^Department of Clinical Laboratory Sciences and Medical Biotechnology, National Taiwan University Hospital, Taipei, Taiwan; ^3^DOE Joint Genome Institute, Walnut Creek, CA, United States; ^4^Department of Veterinary Medicine, National Chung Hsing University, Taichung, Taiwan; ^5^The Department of Nursing, Shu-Zen Junior College of Medicine and Management, Kaohsiung, Taiwan; ^6^Rong Hsing Research Center for Translational Medicine, College of Life Sciences, National Chung Hsing University, Taichung, Taiwan; ^7^Division of Infectious Diseases, Department of Internal Medicine, Taichung Veterans General Hospital, Taichung, Taiwan

**Keywords:** genome sequencing, *Stenotrophomonas acidaminiphila*, sulfamethoxazole resistance, *Stenotrophomonas*, comparative genomics, dihydropteroate synthase

## Abstract

*Stenotrophomonas acidaminiphila* is an aerobic, glucose non-fermentative, Gram-negative bacterium that been isolated from various environmental sources, particularly aquatic ecosystems. Although resistance to multiple antimicrobial agents has been reported in *S. acidaminiphila*, the mechanisms are largely unknown. Here, for the first time, we report the complete genome and antimicrobial resistome analysis of a clinical isolate *S. acidaminiphila* SUNEO which is resistant to sulfamethoxazole. Comparative analysis among closely related strains identified common and strain-specific genes. In particular, comparison with a sulfamethoxazole-sensitive strain identified a mutation within the sulfonamide-binding site of *folP* in SUNEO, which may reduce the binding affinity of sulfamethoxazole. Selection pressure analysis indicated *folP* in SUNEO is under purifying selection, which may be owing to long-term administration of sulfonamide against *Stenotrophomonas*.

## Introduction

Bacteria within the genus *Stenotrophomonas* species are aerobic, glucose non-fermentative, Gram-negative bacilli which inhabit diverse marine and terrestrial environments. The genus *Stenotrophomonas* currently comprises of 14 species^1^. *Stenotrophomonas acidaminiphila* was identified in 2002 (Assih et al., 2002). Initially isolated from sewage sludge from wastewater treatment, it isolated mostly from aquatic environments. Reports of *S. acidaminiphila* human isolates are limited. To our best knowledge, no case of *S. acidaminiphila* infections has ever been reported to date. However, studies of environmental isolates revealed highly resistant to multiple antibiotics (Assih et al., 2002; Vinuesa and Ochoa-Sanchez, 2015).

The antimicrobial options for *Stenotrophomonas* infections are limited because of its inherent resistance to most antibiotics, where trimethoprim-sulfamethoxazole (trimethoprim and sulfonamide combination in a 1:5 ratio) has long been regarded as the agent of choice (Sanchez, 2015). The main component, sulfamethoxazole interrupts the biosynthesis of tetrahydrofolic acid in both bacteria and primitive eukaryotes by targeting the dihydropteroate synthase (DHPS) catalyses, which catalyzes the condensation of 6-hydroxymethyl-7,8-dihydropterin monophosphate (DHPP) with p-aminobenzoic acid (PABA) (Skold, 2000). However, the resistance to sulfamethoxazole is increasing and is mainly caused by single amino acid mutations in the chromosomal gene encoding DHPS or by the acquisition of *sul* genes encoding alternative drug-resistance variants of the DHP via mobile genetic elements (Toleman et al., 2007).

To date, only two environmental strains of *S. acidaminiphila* genomes have been sequenced, all of which were isolated from river sediments (Assih et al., 2002; Vinuesa and Ochoa-Sanchez, 2015). However, the genome, pathogenome, and antimicrobial resistome of clinical isolate can still differ a lot in comparison with those of environmental strains, owing to the adaptation to host immune system and antibiotic pressure. Therefore, a complete genome from clinical isolates is valuable for designing effective treatment strategies.

Here, we sequenced the genome of the *S. acidaminiphila* strain SUNEO, a first clinical isolate that possessed trimethoprim-sulfamethoxazole resistance. We propose a scenario for the origin and evolution of *S. acidaminiphila* SUNEO, based on its genomic features. Gene annotation and comparative analysis further revealed a unique profile of *folP* mutation that could play a role in drug resistance.

## Materials and Methods

### Bacterial Strain Isolation, Identification, and Antimicrobial Susceptibility Testing

Strain SUNEO was isolated from the bile of a cholangiocarcinoma patient with obstructive jaundice and cholangitis. The bile sample was inoculated on trypticase soy agar with 5% sheep blood (Becton–Dickinson, Franklin Lakes, NJ, United States) and incubated aerobically at 37°C overnight. The isolate was identified through 16S rRNA gene sequencing as previously described (Assih et al., 2002; Mangwani et al., 2014). Antibiotic susceptibility tests for the strain SUNEO was performed by the automated Vitek 2 system (bioMérieux, Inc., Durham, NC, United States) according to the manufacturer’s instructions.

### Library Preparation and Whole-Genome Sequencing

Overnight cultures were grown in Luria-Bertani broth overnight at 37°C. Genomic DNA was extracted using DNeasy blood and tissue kit (Qiagen, Valencia, CA, United States) as per the manufacturer’s instructions. High-molecular-weight gDNA was sheared to 10-kb lengths using g-TUBES (Covaris, Woburn, MA, United States). Sheared DNA was processed into PacBio sequencing library. DNA damage repair, end repair, and ligation of SMRT adapters were performed using PacBio SMRTbell Template Prep Kit (Pacific Biosciences). Whole genome sequencing was performed using PacBio sequencing platform (Pacific Biosciences, Menlo Park, CA, United States). Sequence runs of three single-molecule real-time (SMRT) cells were performed on the PacBio RS II sequencer with a 120-min movie time/SMRT cell. SMRT Analysis portal version 2.1 was used for read filtering and adapter trimming, with default parameters, and post-filtered data of 1.479 Gb (∼404X coverage) with an average read length of ∼6.2 kb was used for subsequent assembly (Supplementary Table 1).

### Genome Assembly and Gene Annotation

The post-filtered genome reads were *de novo* assembled by Canu (v1.4) (Koren et al., 2017), which produced one single large contig (∼3.6 Mb). Circlator was used to circularize this contig into a complete circular genome (Hunt et al., 2015). Protein-coding and non-coding genes in the SUNEO genome were annotated using National Center for Biotechnology Information (NCBI) Prokaryotic Genomes Automatic Annotation Pipeline (PGAAP). Functional classification of these annotated genes was carried out by RPSBLAST version 2.2.15 in conjunction with Clusters of Orthologous Groups (COGs) of proteins databases (*E*-values < 0.001).

### Comparative Genomics Analysis and Classification of Pan-Genomic Core Genes and Strain-Specific Genes

To study the comparative genomics of *S. acidaminiphila*, three whole genome sequences of *S. acidaminiphila* strains; *S. acidaminiphila* SUNEO, JCM 13310 (Assih et al., 2002), and ZAC14D2_NAIMI4_2 (Vinuesa and Ochoa-Sanchez, 2015) were downloaded from the NCBI database (**Table 1**). The protein sequences of all three strains were BLAST-aligned against each other (*E*-value < 0.001). However, BLAST may identify false homologs due to repeat sequences commonly shared by multiple genes. Thus, a gene is considered to be shared by both strains if the alignment coverage of both genes is at least 60%. The cutoff was determined by the statistics of alignment coverage of all gene-pairs. We observed that 60% act as a good cutoff for balancing sensitivity and specificity. We consider each gene to be strain-specific if it is presented in only one strain and lost in all other strains. On the other hand, the genes shared by all strains are considered to be pan-genomic core genes.

**Table 1:** Overview of the *S. acidaminiphila* strains in this study.

Strain	Site of isolation	Country of origin	Reference
SUNEO	Human bile	Taiwan	This study
ZAC14D2_NAIMI4_2	Superficial sediments of polluted river	Mexico	Vinuesa and Ochoa-Sanchez, 2015
JCM 13310	Sludge from anaerobic chemical waste water reactor	Mexico	Assih et al., 2002

### 16S rRNA Phylogenetic Analysis

The publicly available 16S rRNA sequences of type strains of *Stenotrophomonas* spp. (including the *S. acidaminiphila* strain SUNEO) were retrieved from the National Center for Biotechnology Information (NCBI) nucleotide database (Supplementary Table 2) (Pruitt et al., 2007; Alavi et al., 2014; Davenport et al., 2014; Patil et al., 2016). In particular, two *S. acidaminiphila* strains (i.e., JCM 13310 and ZAC14D2 NAIMI4) were included in order to confirm the phylogenetic status of SUNEO. At first, multiple sequence alignment of the 16S rRNA gene sequences of all strains was first performed by MEGA (v7). Specifically, ClustalW was used for multiple sequence alignment. Evolution history was reconstructed using the built-in maximum-likelihood method with 1,000 bootstraps.

### Multi-Locus Sequence Typing Using Multiple Housekeeping Genes

To further validate these clade assignments, multilocus sequence analysis (MLSA) was performed by concatenation of housekeeping genes: *atpD, guaA, mutM, nuoD, ppsA*, and *recA* (Kaiser et al., 2009). Multiple sequence alignment of these housekeeping genes in 15 *Stenotrophomonas* genomes was performed using MEGA in order to infer their phylogeny (Alavi et al., 2014; Davenport et al., 2014; Patil et al., 2016; Sanchez-Castro et al., 2017). Two conventional *gyrB* and *gapA* were not included because *gyrB* is completely absent in *S. acidaminiphila* JCM 13310 and only very short piece of *gapA* is found in *S. ginsengisoli* DSM 24757. Additional MLSA using the six housekeeping genes plus 16S rRNA is also performed using MEGA (v7) to confirm the phylogenetic position of SUNEO.

### Whole-Genome Average Nucleotide Identity Analysis

To measure the nucleotide-level genomic similarity between SUNEO and related *Stenotrophomonas* genomes (Alavi et al., 2014; Davenport et al., 2014; Patil et al., 2016), the Average Nucleotide Identity (ANI) (Konstantinidis and Tiedje, 2005) was calculated by the USEARCH program (Yoon et al., 2017) based on modified OrthoANI algorithm (Lee et al., 2016). A radial phylogram was constructed using distance matrix computations using the Integrated Microbial Genomes pipeline (Chen et al., 2017).

### Annotation of Antibiotic-Resistance Genes

The SUNEO resistome is annotated by using both the Resistance Gene Identifier from the Comprehensive Antibiotic Resistance Database (McArthur et al., 2013) and the IMG database (Markowitz et al., 2012). RGI prediction of resistome is based on homology and SNP models, where the strict criteria were chosen for prediction. In homolog models, BLAST is used to detect functional homologs of antimicrobial resistance genes. In contrast, SNP models identify candidate genes which acquire mutations conferring antimicrobial resistance genes based on curated SNP matrices. The SUNEO resistome is predicted through alignment against the IMG database using BLASTN with a 95% sequence identity threshold.

### Sequence, Structural, and Selection Analysis of *folP* Gene

The phylogeny of the DHPS protein was constructed by MEGA7. The publicly available *folP* homolog gene sequences of 18 *Stenotrophomonas* strains (including *S. acidaminiphila* strain SUNEO) were retrieved from the National Center for Biotechnology Information (NCBI) nucleotide database (Supplementary Table 3). The amino acid sequences of DHPS from SUNEO and JCM 13310 were BLAST-aligned against each other in order to identify the mutation loci and annotate the conservative loop regions. To probe its topological structure, the 3D structure of DHPS proteins were predicted by Robetta^2^, and subsequently visualized by NOC 3.01^3^. In conjunction with the annotated loop regions in the sequence level, we were able to compare the local substructure of Loop2 between the two strains. The ratio of non-synonymous to synonymous substitutions (i.e., Ka/Ks) is used to estimate positive and purifying selection at each amino-acid site in *folP* for SUNEO, JCM 13310, and ZAC14D2_NAIMI4_2. The M8 site model (with ω allowed to > 1) with intermediate precision level (𝜀 = 0.1, which defines when two likelihood values converged) is used to compute the Ka/Ks ratio^4^.

## Results

### Genome Overview and Annotation

**FIGURE 1:**
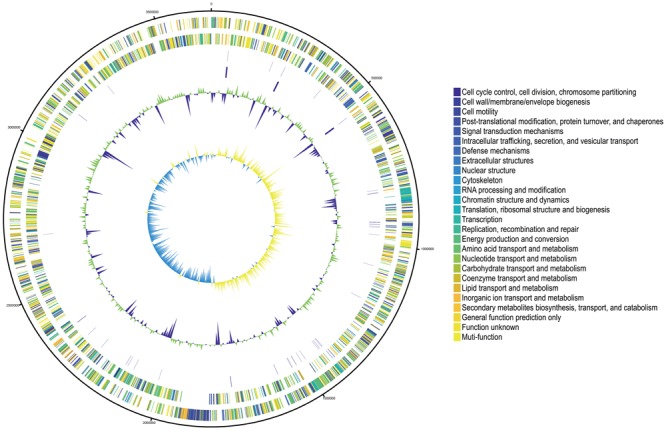
Circular representation of the *S. acidaminiphila* strain SUNEO genome. Predicted Coding Sequences (CDSs) are assigned various colors with respect to cellular functions. The Circles display from the outermost to the innermost: (1) DNA coordinates. (2,3) Function-based color coded mapping of the CDSs predicted on the forward and reverse strands, respectively. (4,5) tRNA and rRNA genes, respectively. (6) GC plot with regions above and below average in green and violet, respectively. (window size: 10,000 bp). (7) GC skew showing regions above and below average in yellow and light blue, respectively. (window size: 10,000 bp).

The total size of the genome is 3,660,864 bp with a G+C content of 69.8%. An illustration of the genomic contents in the genome of SUNEO is shown in **Figure 1**. A total of 3,173 Coding Sequences (CDSs) were predicted (**Table 2**). In addition, 70 RNAs including rRNA and tRNA were identified. No extrachromosomal elements were found in SUNEO.

**Table 2:** Genomic features of *S. acidaminiphila* strain SUNEO, ZAC14D2_NAIMI4_2, and JCM 13310.

Strain	Genome assembly status	Genome size (bp)	GC content (%)	Total Genes^∗ 1^	Total CDS^∗ 2^	Pseudo genes	Total proteins	rRNA	tRNA	Modify date
SUNEO	Complete	3,660,864	69.8	3,247	3,173	292	2,881	9	61	16 February, 2017
ZAC14D2_NAIMI4_2	Complete	4,138,297	68.5	3,709	3,635	65	3,570	9	61	11 April, 2017
JCM 13310	Draft	3,942,520	68.8	3,636	3,573	131	3,442	3	56	11 April, 2017

*^∗*1*^Total genes = Total CDS + rRNA + tRNA + ncRNA. ^∗*2*^Total CDS = Total Proteins + Pseudo genes.*

### Comparative Genomic Study and Identification of Core and Strain-Specific Genes of *S. acidaminiphila* Genomes

General genomic features of *S. acidaminiphila* SUNEO were compared to the *S. acidaminiphila* JCM 13310 and ZAC14D2_NAIMI4_2 (**Table 2**). The genome size of the *S. acidaminiphila* strain ZAC14D2_NAIMI4_2 was the largest (4,138,297 bp) amongst all genomes (ranging from 3,660,864 to 3,942,520 bp). The highest genomic G+C content (69.8%) was from the *S. acidaminiphila* strain SUNEO followed by the *S. acidaminiphila* strain JCM 13310 (68.8%), and the *S. acidaminiphila* strain ZAC14D2_NAIMI4_2 (68.5%).

**FIGURE 2:**
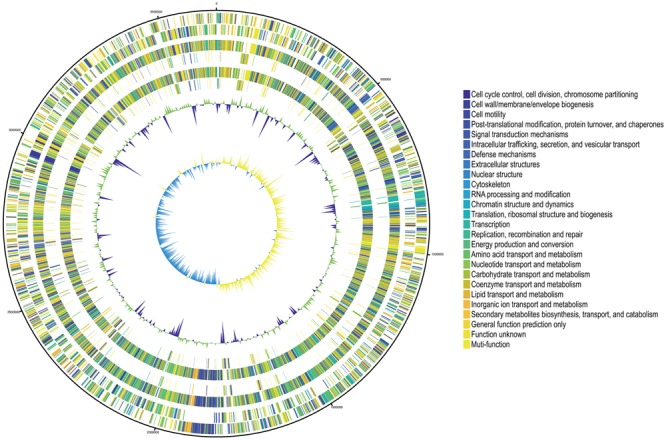
Comparative genome representation of *S. acidaminiphila* strain SUNEO, JCM 13310, and ZAC14D2_NAIMI4_2. Predicted Coding Sequences (CDSs) are assigned various colors with respect to cellular functions. The Circles display from the outermost to the innermost: (1) DNA coordinates. (2,3) Function-based color coded mapping of the CDSs predicted on the forward and reverse strands, respectively. (4) Orthologous CDSs shared between *S. acidaminiphila* strain SUNEO and *S. acidaminiphila* strain JCM 13310. (5) Specific CDSs in *S. acidaminiphila* strain SUNEO, compared with *S. acidaminiphila* strain JCM 13310. (6) Orthologous CDSs shared between *S. acidaminiphila* strain SUNEO and *S. acidaminiphila* strain ZAC14D2_NAIMI4_2. (7) Specific CDSs in *S. acidaminiphila* strain SUNEO, compared with *S. acidaminiphila* strain ZAC14D2_NAIMI4_2. (8) GC plot with regions above and below average in green and Violet, respectively. (window size: 10,000 bp). (9) GC skew showing regions above and below average in yellow and light blue, respectively. (window size: 10,000 bp).

**FIGURE 3:**
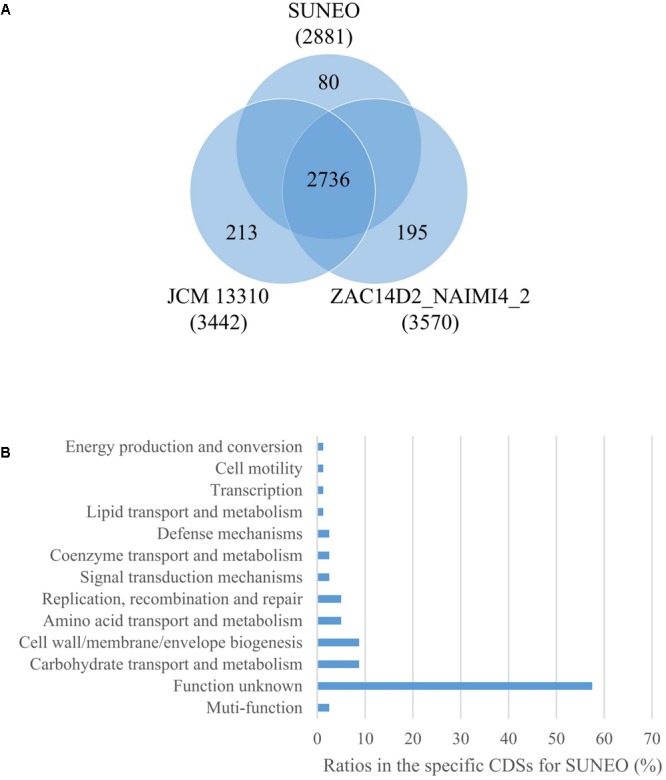
Comparison of the gene contents among *Stenotrophomonas acidaminiphila* strain SUNEO, JCM 13310, and ZAC14D2_NAIMI4_2. **(A)** Partial Venn diagram showing the numbers of shared (core) and strain-specific CDSs. **(B)** Diagram showing the COG category-based functional analysis of SUNEO-specific CDSs.

The protein-coding genes of SUNEO were compared with those of *S. acidaminiphila* JCM 13310 and ZAC14D2_NAIMI4_2, in order to identify orthologous core genes which are shared across all strains and strain-specific genes. **Figure 2** depicts both the positions and the color-coded functions of *S. acidaminiphila* SUNEO genes in comparison with all other strains, whereas the number of orthologous and strain-specific genes is shown in **Figure 3**. In summary, the core genome of *S. acidaminiphila* consisted of 2,736 core genes shared across all strains, whereas 807 genes are specific only to *S. acidaminiphila* SUNEO (**Figure 3A**). Functional analysis of SUENO-specific genes revealed that, in addition to hypothetical proteins, a relative abundance of the gene is involved in carbohydrate transport, along with metabolism and cell wall/membrane/envelop biogenesis (**Figure 3B**). DHPS encoding *folP* homologs genes are presented in all strains.

### Phylogenetic Analysis of *S. acidaminiphila*

A maximum-likelihood tree of the three *S. acidaminiphila* genomes and 12 reported *Stenotrophomonas* strains (comprising of *S. daejeonensis, S. humi, S. pictorum, S. terrae, S. nitritireducens, S. ginsengisoli, S. koreensis, S. maltophilia, S. pavanii, S. chelatiphaga, S. panacihumi*, and *S. rhizophila*) was created based on 16S rRNA gene sequences (Supplementary Figure 1). This phylogenetic tree shows *S. acidaminiphila* SUNEO, JCM 13310, and ZAC14D2_NAIMI4_2 grouped together. The result was further supported by Alignment Fraction analysis, which showed SUNEO was included in the *S. acidaminiphila* JCM 13310 and ZAC14D2_NAIMI4_2 phylogenetic subgroup (Supplementary Figure 2).

**FIGURE 4:**
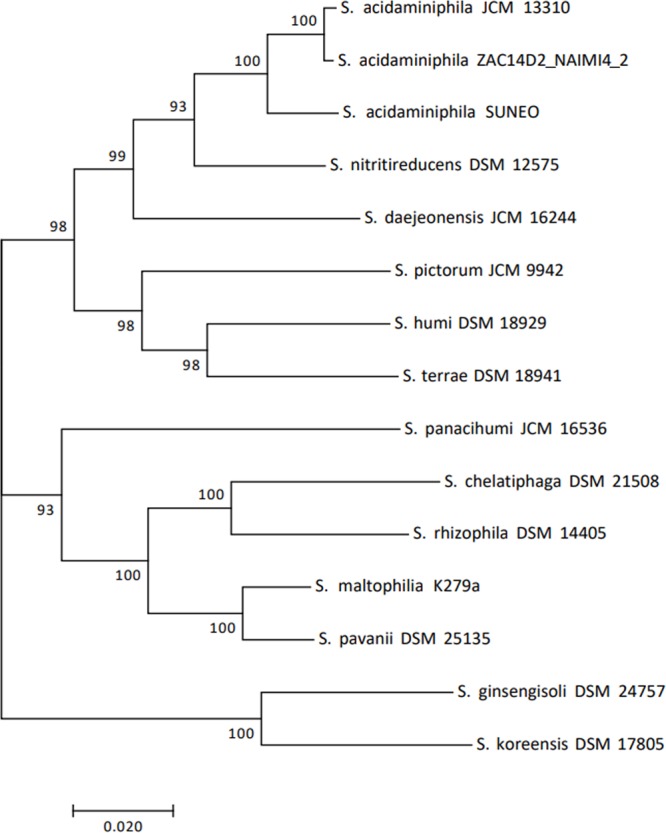
Phylogenetic tree of *S. acidaminiphila* SUNEO and related type strains of *Stenotrophomonas* species based on the phylogenetic analysis of seven housekeeping genes (16S rRNA, *atpD, guaA, mutM, nuoD, ppsA*, and *recA*).

The MLSA using conventional housekeeping genes (with or without 16S RNA) both revealed high phylogenetic similarities higher than 99% among SUNEO, JCM 13310, and ZAC14D2_NAIMI4_2 (**Figure 4** and Supplementary Figure 3), which was the accepted species threshold (Kaiser et al., 2009). Genomic-wide relatedness comparison was calculated with the OrthoANI program using publicly available genomes from type strains of *Stenotrophomonas* species (Alavi et al., 2014; Davenport et al., 2014; Patil et al., 2016; Sanchez-Castro et al., 2017). As the ANI value of SUNEO to *S*. *acidaminiphila* strain is 92.94–92.83% (Supplementary Table 4), indicated a taxonomic outlier (Gomila et al., 2015). Together, all these analysis (from single gene, multiple genes, to entire genome) concordantly concluded that the phylogenetic position of SUNEO is indeed belonging to *S*. *acidaminiphila*.

### Comparative Analysis of Antibiotic Resistance Genes in SUNEO

Antimicrobial susceptibility test showed that SUNEO is resistant to both imipenem and trimethoprim/sulfamethoxazole (**Table 3**). Comparative analysis was performed on the three *S. acidaminiphila* genomes, among which JCM 13310 is trimethoprim/sulfamethoxazole susceptible (**Table 3**). All three strains harbor similar resistance genes. There are Ambler class B β-lactamase L1 and Ambler class A β-lactamase L2 in all of the three strains. The resistance-nodulation-division (RND) family efflux pump, consisting of the *smeDEF* and *smeOP* genes, along with the efflux pumps genes that are homologous to efflux pumps encoded in *S. maltophilia* and *Escherichia coli* were also identified in all of the tested strains (**Table 4**).

**Table 3:** Antimicrobial susceptibility profiles of *S. acidaminiphila* SUNEO and JCM 13310.

Class	Subclass	Antibiotics	SUNEO^∗^	JCM 13310^∗^
Aminoglycoside antibiotic	–	Amikacin	16, S	≤ 8, S
		Gentamicin	2, S	≤ 4, S
		Netilmicin		≤ 4, S
		Tobramycin		≤ 4, S
Fluoroquinolone antibiotic	–	Ciprofloxacin	≤ 0.25, S	≤ 1, S
		Ofloxacin		≤ 1, S
Lipopeptide antibiotic	Polymyxin antibiotic	Colistin		≤ 2, S
Diaminopyrimidine antibiotic Sulfonamide antibiotic	–	Trimethoprim Sulfamethoxazole	80(4/76), R	≤ 2/38, S
β-lactam antibiotic	Carbapenem	Imipenem	≥ 16, R	> 8, R
	Cephem	Cefalotin		> 32, R
		Cefepime	≤ 1, S	
		Cefotaxime		> 32, R
		Ceftriaxone	16, I	
		Ceftazidime	≤ 1, S	≤ 4, S
	Penam	Amoxicillin		> 16, R
		Piperacillin		≤ 16, S
		Ticarcillin		≥ 16–64, I
β-lactam combination agents	Penam β-lactamase inhibitor	Amoxicillin Clavulanic acid		> 16, R
		Ampicillin Sulbactam	≤ 2, S	
		Piperacillin Tazobactam	≤ 4, S	≤ 16, S
Tetracycline derivative	Glycylcycline	Tigecycline	≤ 0.5, S	

*^∗^R, resistant; S, susceptible; I, intermediate.*

**Table 4:** Summary of the antibiotic resistance genes among the three strains of *S. acidaminiphila* and their related locus tag.

	SUNEO	ZAC14D2_NAIMI4_2	JCM 13310
**β-lactam resistance gene**			
Class A β-lactamase L2	B1L07_04670	AOT14_RS05350	ABB33_RS08520
Class B metallo-β-lactamase L1	B1L07_11340	AOT14_RS12805	ABB33_RS04665
VEB β-lactamase			
VEB-5	B1L07_15655	AOT14_RS07520	ABB33_RS15710
**Aminoglycoside resistance gene**			
AAC(6′)			
AAC(6′)-Ic	B1L07_09660	AOT14_RS11055	ABB33_RS07955
**Fluoroquinolone resistance gene**			
Quinolone resistance gene			
QnrB27	B1L07_15000	AOT14_RS17045	ABB33_RS01740
**Efflux pump**			
**RND efflux pump**			
**AcrAB-TolC RND system**			
acrR	B1L07_02745	AOT14_RS03270	ABB33_RS01685
acrA	B1L07_02750	AOT14_RS03275	ABB33_RS01690
acrB	B1L07_02755	AOT14_RS03280	ABB33_RS01695
**SmeDEF RND system**			
smeD	B1L07_07555	AOT14_RS08230	ABB33_RS10360
smeE	B1L07_07560	AOT14_RS08235	ABB33_RS10365
smeF	B1L07_07570	AOT14_RS08245	ABB33_RS10375
**SmeOP-TolC RND system**			
smeO	B1L07_03315	AOT14_RS03840	ABB33_RS09950
smeP	B1L07_03320	AOT14_RS03845	ABB33_RS09945
**MFS efflux pump**			
**NorA MFS system**			
norA	B1L07_03630	ABB33_RS16160	AOT14_RS06550
arlR	B1L07_05940	ABB33_RS10760	AOT14_RS06670
arlS	B1L07_05945	ABB33_RS10755	AOT14_RS06675
mgrA	B1L07_12295	ABB33_RS03745	AOT14_RS13715
**EmrAB-TolC MFS system**			
emrA	B1L07_10580	AOT14_RS12025	ABB33_RS05915
emrB	B1L07_10575	AOT14_RS12020	ABB33_RS05920
tolC	B1L07_03300	AOT14_RS03825	ABB33_RS09965

### Phylogeny of *folP* Homologs in *Stenotrophomonas* Strains

**FIGURE 5:**
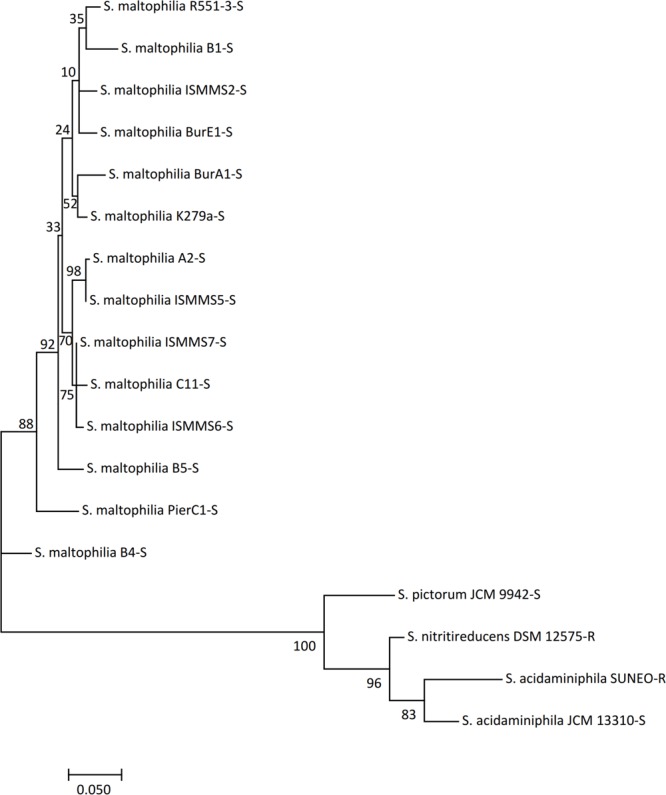
Phylogenetic tree of the amino acid sequences of dihydropteroate synthase homologs from *Stenotrophomonas* strains. Evolutionary history was inferred by using the maximum likelihood method. The bootstrap consensus tree inferred from 1,000 replicates is taken to represent the evolutionary history of the taxa analyzed. R: resistant to TMP-SMX. S: susceptible to TMP-SMX.

Comparative genomic analysis has revealed the gene *folP* is commonly presented among *S. maltophilia* strains. *folP* encodes DHPS and is the target of sulfonamides, to which SUNEO is resistant. A BLASTP search in *Stenotrophomonas* strains with *folP* as the primer sequence returned a collection of related homologs with the annotation of DHPS (Baker and Sali, 2001). We retrieved 18 protein sequence candidates with available MIC data for each strain. The phylogeny of the DHPS protein constructed by MEGA7 (Kumar et al., 2016) clearly presented two distinct groups: one denotes a family of *folP* homologs from *S. maltophilia* whereas the other comprises a series of *folP* homologs from non-maltophilia *Stenotrophomonas* strains (**Figure 5**). Of particular note, *folP* homologs between SUNEO and JCM 13310 are highly similar in comparison with other distant-related strains. This implies that the resistance of SUNEO to sulfonamides is due to few/key mutations acquired occasionally instead of continual accumulation of resistant alleles after speciation.

### Sulfonamide-Binding Site Mutation Revealed by Structural Analysis

**FIGURE 6:**
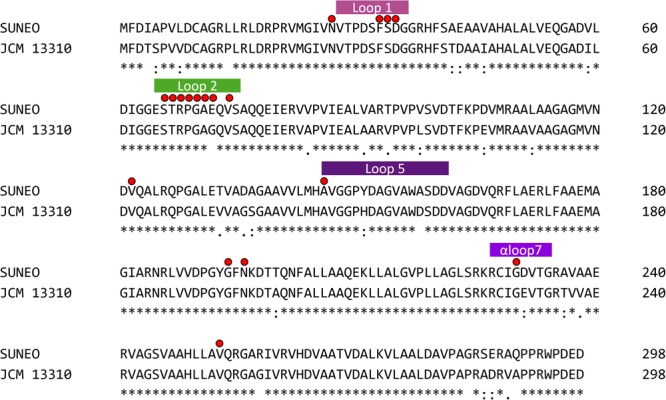
Alignment result of the *folP*s in the *S. acidaminiphila* strain SUNEO and JCM 13310. Residues marked with red dots are sites of common sulfa drug (sulfonamide) resistance mutations. The last line in the data means the consensus amino acid symbol of the residue in the corresponding position for all strains: an asterisk indicates positions which have a single, fully conserved residue; a colon indicates conservation between groups of strongly similar properties; a period indicates conservation between groups of weakly similar properties.

**FIGURE 7:**
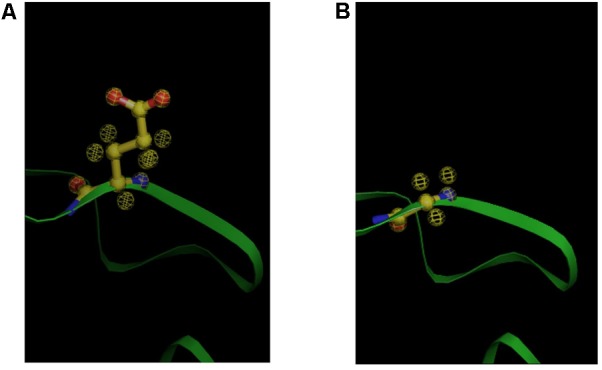
Structure predication for the *folP* gene products (dihydropteroate synthase) of *S. acidaminiphila* SUNEO and JCM 13310. **(A)** Glu72 in the SUNEO dihydropteroate synthase. **(B)** Gly72 in the JCM 13310 dihydropteroate synthase.

In order to identify key mutation in *folP* leading to resistance of SUNEO, sequence composition of DHPS between JCM 13310 and SUNEO was compared, which exhibited an amino acid change (Gly^72^ → Glu^72^) in one highly conservative region (termed Loop2) (**Figure 6**). Structural modeling allows us to visualize the difference in DHPS architecture between these two strains. This conservative region stabilizes the binding of PABA and variation at this region has been shown to contribute to resistance to sulfonamide (Yun et al., 2012). We further reconstruct their protein 3D structures to verify the difference of DHPS architecture between these two strains. The protein substructure at Loop2 in SUNEO appears disordered in comparison with that of JCM 13310 (**Figure 7**), which may reduce the binding stability of sulfonamide and lead to resistance.

**FIGURE 8:**
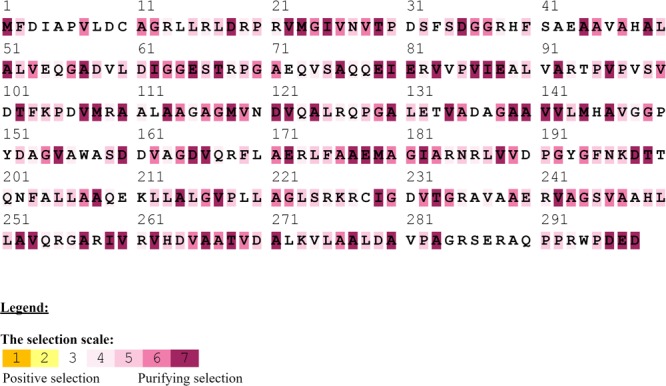
Selection pressure (Ka/Ks) measured along *folP* among *S. acidaminiphila* strains. The strong signals of purifying selection are defined as the loci with Ka/Ks < 0.8. Overall, 72 loci of strong signals of purifying selection is wide-spread across the entire gene sequence.

The selection pressure (Ka/Ks) was measured along the entire *folP* (**Figure 8**). The results indicated that strong signals of purifying selection (Ka/Ks < 0.08) are widely spread in *folP*. This implies evolution of *folP* in SUNEO is probably constrained by high selection pressure from long-term exposure of sulfonamide.

## Discussion

The data we present represents a first glimpse into the evolution and mechanism for sulfamethoxazole resistance in *S. acidaminiphila*. Our discovery of novel mutations in DHPS offers new insight into the newly emerging field of *Stenotrophomonas* infections, furthering our understanding of the diversity in the dissemination of sulfamethoxazole resistance. Sulfamethoxazole interrupt the essential folate pathway in bacteria by targeting the enzyme DHPS, which in turn catalyzes the condensation of DHPP with PABA in the production of the folate intermediate. The locus of the mutation is consistent with earlier observations that many sulfamethoxazole resistance mutations are located within two conserved loops (called Loop1 and Loop2) of DHPS, which creates a specific binding pocket for PABA (Estrada et al., 2016).

To gain further structural insight of the DHPS homologs in SUNEO and JCM 13319, structural modeling was performed, where the ribbon structures of their DHPS were generated. The 3D structural DHPS proteins in SUNEO illustrated that the substructure at Loop2 is disordered. Further structural comparisons of DHPS also indicated that the resistant (SUNEO) and sensitive (JCM 13310) strains display a different substructure around the PABA-binding pockets. In agreement with the earlier proposal by Yun et al. (2012), sulfonamide resistance is associated with the Loop2 mutation and subsequent DHPS substructure disorder affected the binding of PABA. It was also reported that mutations in DHPS were associated with sulfamethoxazole resistance in both prokaryotes (Huovinen, 2001) and primitive eukaryotes (Triglia et al., 1997), where associated structural changes in Loop1–Loop2 PABA binding sites occurred (Capasso and Supuran, 2014). As sulfa drugs interrupt the folate pathway by competing with PABA as DHPS substrates, mutation at both the sequence and the structure of DHPS in SUNEO could be attributed to its resistance to sulfamethoxazole.

The selection pressure measured along the DHPS-encoding gene *folP* reveals strong signals of purifying selection, implying the evolution of *folP* is constrained by high selection pressure. Sulfamethoxazole has been the first-line antibiotic agent against *Stenotrophomonas* for decades and is widely use in agriculture. The usage of specific antibiotic agent has been shown to result in purifying selection in certain genes of resistant strains (Mortimer et al., 2018), because purifying selection on a subset of genes can be intensified in the setting of resistance. After sufficiently long-term usage of the same antibiotic agent, resistance may even reach a point of stabilizing evolution, completely reducing or invaliding the efficiency of the drug (Cornick et al., 2014). As *folP* in *S. acidaminiphila* already exhibited purifying selection, the administration of sulfamethoxazole for *S. acidaminiphila* infections in the future should be taken with caution.

*Stenotrophomonas acidaminiphila* SUNEO is also resistant to various antibiotics, suggesting multiple resistance mechanisms. Current understanding of the resistance determinants of *S. acidaminiphila* is limited. Vinuesa and Ochoa-Sanchez (2015) reported on predicted antibiotic resistant genes (without phenotypic resistance) in *S. acidaminiphila* ZAC14D2_NAIMI4_2 isolated from river sediment in Mexico, while Assih et al. (2002) reported on phenotypic resistance (without genotypic changes) in *S. acidaminiphila* JCM 13310. To address this concern, we conducted resistome analysis of *S. acidaminiphila* SUNEO and predicted multiple efflux pumps, which have also been detected in *S. maltophilia* strains (Crossman et al., 2008). In particular, we identified the RND efflux pump genes *smeDEF*, which was associated with trimethoprim/sulfamethoxazole resistance (Sanchez and Martinez, 2015). The resistance of trimethoprim/sulfamethoxazole in SUNEO could be a combination effect related to DHPS mutation and efflux pumps.

*Stenotrophomonas acidaminiphila* is able to degrade a number of organic pollutants, including Fomesafen [5-(2-chloro-4-[trifluoromethyl]phenoxy)-N-methylsulfonyl-2-nitrobenzamide] (Huang et al., 2017), Diuron [3-(3,4-dichlorophenyl)-1,1-dimethylurea] (Egea et al., 2017), and azo dye crystal violet (Kim et al., 2002). Our comparative analysis of resistome in *S. acidaminiphila* revealed that the efflux pumps genes presented in all examined *S. acidaminiphila* strains. Efflux pumps play a major role in both solvent tolerance and bioremediation (Fernandes et al., 2003), which is consistent with recent observations of the biodegradation of sulfonamide (Liao et al., 2016) and aminoglycoside (Selvaraj et al., 2018) in *S. acidaminiphila*. The mechanistic insight we gained further raises the possibility of cross-resistance to both environmental toxic compounds and antibiotics which would have a major impact on the use of disinfectants and disinfecting procedures.

Currently, most of the reported *Stenotrophomonas* infections are caused by *S. maltophilia* which is frequently recovered from clinical samples and is an emerging opportunistic pathogen associated with substantial morbidity and mortality, particularly in immunocompromised patients (Falagas et al., 2009). Incidences of human infection appear to have increased recently, where a variety of clinical syndromes have been described, including pneumonia, bacteremia, and peritonitis (Denton and Kerr, 1998; Sattler et al., 2000). However, biliary tract infections remain uncommon. Our report outlines the first human biliary infection caused by *S. acidaminiphila*. The virulence factors of *S. acidaminiphila* are largely unknown. Our data has revealed RND pump homologs of the *acrAB* in SUNEO. *acrAB* encodes a bile-induced efflux system and is expressed in both animal models and infected patients (Gunn, 2000; Piddock, 2006). Additional studies are required in order to clarify its role in *S. acidaminiphila* pathogenesis.

## Conclusion

Our analysis reveals a possible core genome of *S. acidaminiphila*, along with accessory genomes specific to each strain, providing insights into the resistant potential of the clinical isolate. We propose a scenario for the origin and evolution of *S. acidaminiphila*, based on its genomic features. Gene annotation and comparative analysis further revealed a unique profile of *folP* mutation. The mechanism for sulfonamide resistance in *S. acidaminiphila* SUNEO appears to involve the mutation of the Loop2 region of DHPS, thereby leading to alterations in the structural conformation of the site and the multi-drug efflux pumps.

## Data Availability

This genome project, which includes the raw read data, assembly, and annotation, has been deposited at NCBI/GenBank as BioProject PRJNA374779. The assembly is available under accession CP019797; the version described in this paper is the first version.

## Author Contributions

Y-TH, J-MC, and P-YL designed and coordinated the study and carried out the data analysis. Y-TH, J-MC, and P-YL performed the bioinformatics analysis. Z-YW, B-CH, and P-YL carried out the experiments and interpreted data for the work. Y-TH, Z-YW, RK, and P-YL wrote the manuscript. Y-TH, RK, and P-YL checked and edited the manuscript. All authors have read and approved the manuscript.

## Conflict of Interest Statement

The authors declare that the research was conducted in the absence of any commercial or financial relationships that could be construed as a potential conflict of interest.

## Footnotes

1 http://www.bacterio.net/stenotrophomonas.html

2 http://robetta.bakerlab.org/

3 http://noch.sourceforge.net

4 http://selecton.tau.ac.il
