# Supplementary material for: Genome Sequencing and Comparative Analysis of Stenotrophomonas acidaminiphila Reveal Evolutionary Insights Into Sulfamethoxazole Resistance
Source: Front Microbiol. 2018 May 17;9:1013. doi: 10.3389/fmicb.2018.01013 (PMC5966563; doi:10.3389/fmicb.2018.01013)
Supplement: Supplementary file 1 [file Table_1.DOCX]

Supplementary Table 1. Sequencing statistic of three SMRT cells computed by ABySS-fac

.

| **SMRT Cells** | **No. Reads** | **N80** | **N50** | **N20** | **Max** | **Total bases (bp)** |
| --- | --- | --- | --- | --- | --- | --- |
| 1 | 99,051 | 4,520 | 6,171 | 8,799 | 33,873 | 429,900,000 |
| 2 | 90,523 | 4,439 | 6,071 | 8,662 | 39,640 | 377,600,000 |
| 3 | 98,594 | 4,431 | 6,068 | 8,718 | 38,046 | 413,900,000 |
| Total | 288,168 | - | - | - | - | 1,479,300,000 |
