# Supplementary material for: Genome Sequencing and Comparative Analysis of Stenotrophomonas acidaminiphila Reveal Evolutionary Insights Into Sulfamethoxazole Resistance
Source: Front Microbiol. 2018 May 17;9:1013. doi: 10.3389/fmicb.2018.01013 (PMC5966563; doi:10.3389/fmicb.2018.01013)
Supplement: Supplementary file 3 [file Table_3.DOCX]

| **Accession Number** | **Site of isolation** | **Country of origin** | **Species** | **Strain / Isolation** | **MIC( (mg/liter) to TMP-SMX** | | |
| --- | --- | --- | --- | --- | --- | --- | --- |
| CP019797 | Homo sapiens | Taiwan | *S. acidaminiphila* | **SUNEO** | 80 | R |  |
| NZ_LDJO01000001.1 | Sludge | Mexico |  | **JCM 13310 AMX 19** | ≤ 2/38 | S |  |
| NZ_BAZI00000000.1 | - | - | *S. pictorum* | **JCM 9942 ATCC 23328** | 0.02 | S |  |
| NZ_LDJG00000000.1 | biofilter | Germany | *S. nitritireducens* | **DSM 12575 L2** |  | R |  |
| NZ_CVIW00000000.1 | Surface bulk soil | Burkina Faso | *S. maltophilia* | **BurA1** | 40 | S |  |
| NZ_CVIU00000000.1 |  |  |  | **BurE1** | ≤ 20 | S |  |
| NZ_CVIV00000000.1 |  | France |  | **PierC1** | ≤ 20 | S |  |
| NC_011071.1 | Poplar tree endophyte | USA |  | **R551-3** | ≤ 20 | S |  |
| NC_010943.1 | Homo sapiens | UK |  | **K279a** | ≤ 20 0.125 1/19 | S S S |  |
| NZ_LDVQ00000000.1 |  | Australia |  | **B1** |  | S |  |
| NZ_LDVR00000000.1 |  |  |  | **B4** |  | S |  |
| NZ_LDVS00000000.1 |  |  |  | **B5** |  | S |  |
| NZ_LDVP00000000.1 |  |  |  | **A2** |  | S |  |
| NZ_LDVT00000000.1 |  |  |  | **C11** |  | S |  |
| NZ_CP011305.1 |  | USA |  | **ISMMS2** | <20 0.19 | S S |  |
| NZ_JZIV00000000.1 |  |  |  | **ISMMS5** | <20 0.25 | S S |  |
| NZ_JZIW00000000.1 |  |  |  | **ISMMS6** | <20 1.5 | S S |  |
| NZ_JZTX00000000.1 |  |  |  | **ISMMS7** | <20 1.5 | S S |  |

Supplementary Table 3. TMP-SMX (Trimethoprim/sulfamethoxazole) MIC profiles of *folp*-carrying *Stenotrophomonas* strains. "R" and "S" mean that the strains are resistant and sensitive to TMP-SMX, respectively.
