# Supplementary material for: Genome Sequencing and Comparative Analysis of Stenotrophomonas acidaminiphila Reveal Evolutionary Insights Into Sulfamethoxazole Resistance
Source: Front Microbiol. 2018 May 17;9:1013. doi: 10.3389/fmicb.2018.01013 (PMC5966563; doi:10.3389/fmicb.2018.01013)

Supplementary Material

Genome sequencing and comparisive analysis of Stenotrophomonas acidaminiphila reveal evolutionary insights into sulfamethoxazole resistance

Yao-Ting Huang, Jia-Min Chen, Bing-Ching Ho, Zong-Yen Wu, Rita C. Kuo, Po-Yu Liu

*** Correspondence:** Po-Yu Liu: pyliu@vghtc.gov.tw

Supplementary Figures

Supplementary Figure1. 16S Rrna phylogenetic analysis of *S. acidaminiphila* SUNEO.

A maximum likelihood tree of *Stenotrophomonas* strains from NCBI whole genome shotgun sequences was constructed using MEGA v7.0.26, based on the 16S rRNA sequence in each genome. Bootstrap values (1,000 replications) are shown at the interior branches.

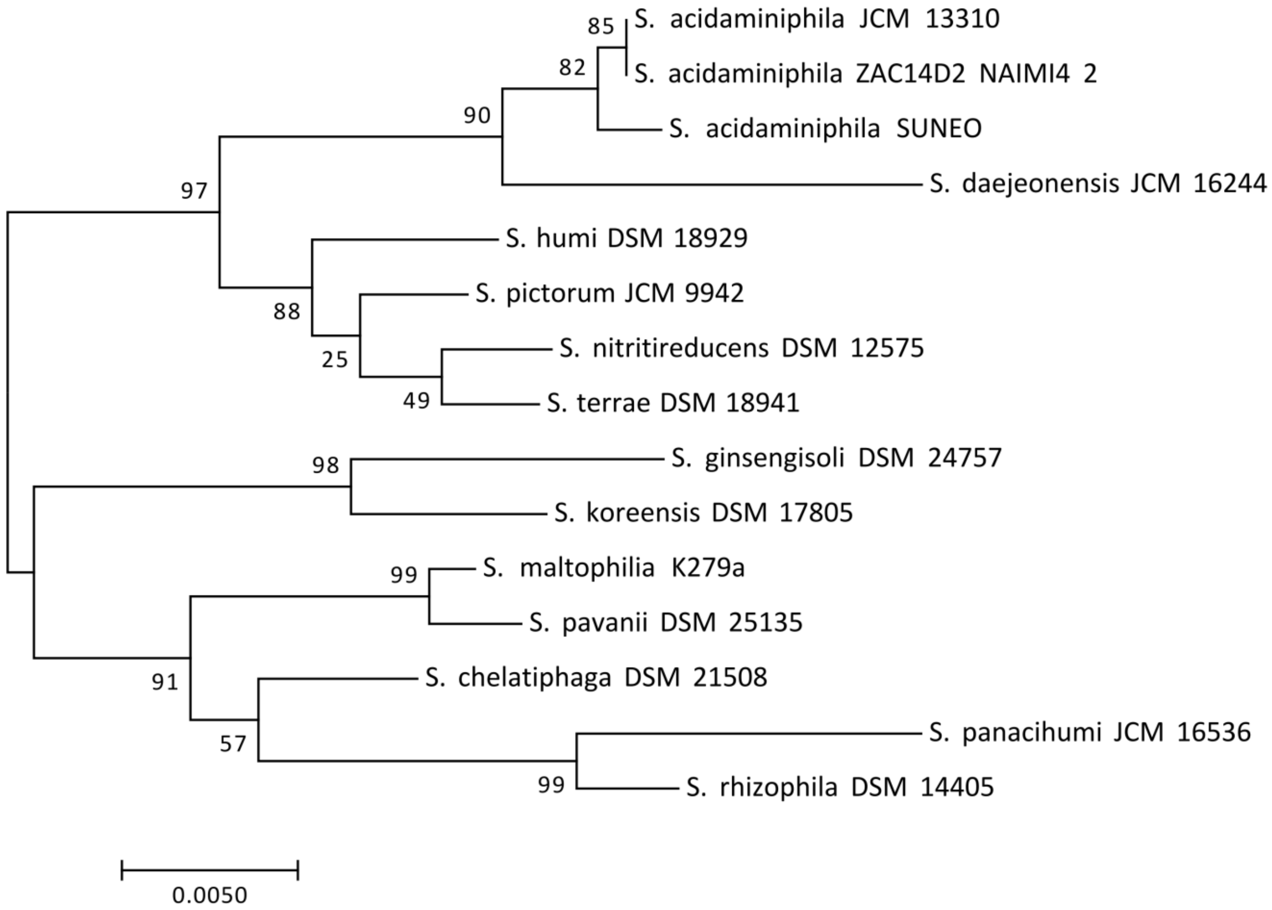

Supplement: Supplementary file 5 [file Image_1.PDF]
