# Supplementary material for: Genome Sequencing and Comparative Analysis of Stenotrophomonas acidaminiphila Reveal Evolutionary Insights Into Sulfamethoxazole Resistance
Source: Front Microbiol. 2018 May 17;9:1013. doi: 10.3389/fmicb.2018.01013 (PMC5966563; doi:10.3389/fmicb.2018.01013)
Supplement: Supplementary file 6 [file Image_2.PDF]

Supplementary Material

Genome sequencing and comparisive analysis of Stenotrophomonas acidaminiphila reveal evolutionary insights into sulfamethoxazole resistance

Yao-Ting Huang, Jia-Min Chen, Bing-Ching Ho, Zong-Yen Wu, Rita C. Kuo, Po-Yu Liu

*** Correspondence:** Po-Yu Liu: pyliu@vghtc.gov.tw

Supplementary Figures

Supplementary Figure2. Radial phylogram based on the genomic data from *Stenotrophomonas* strains.

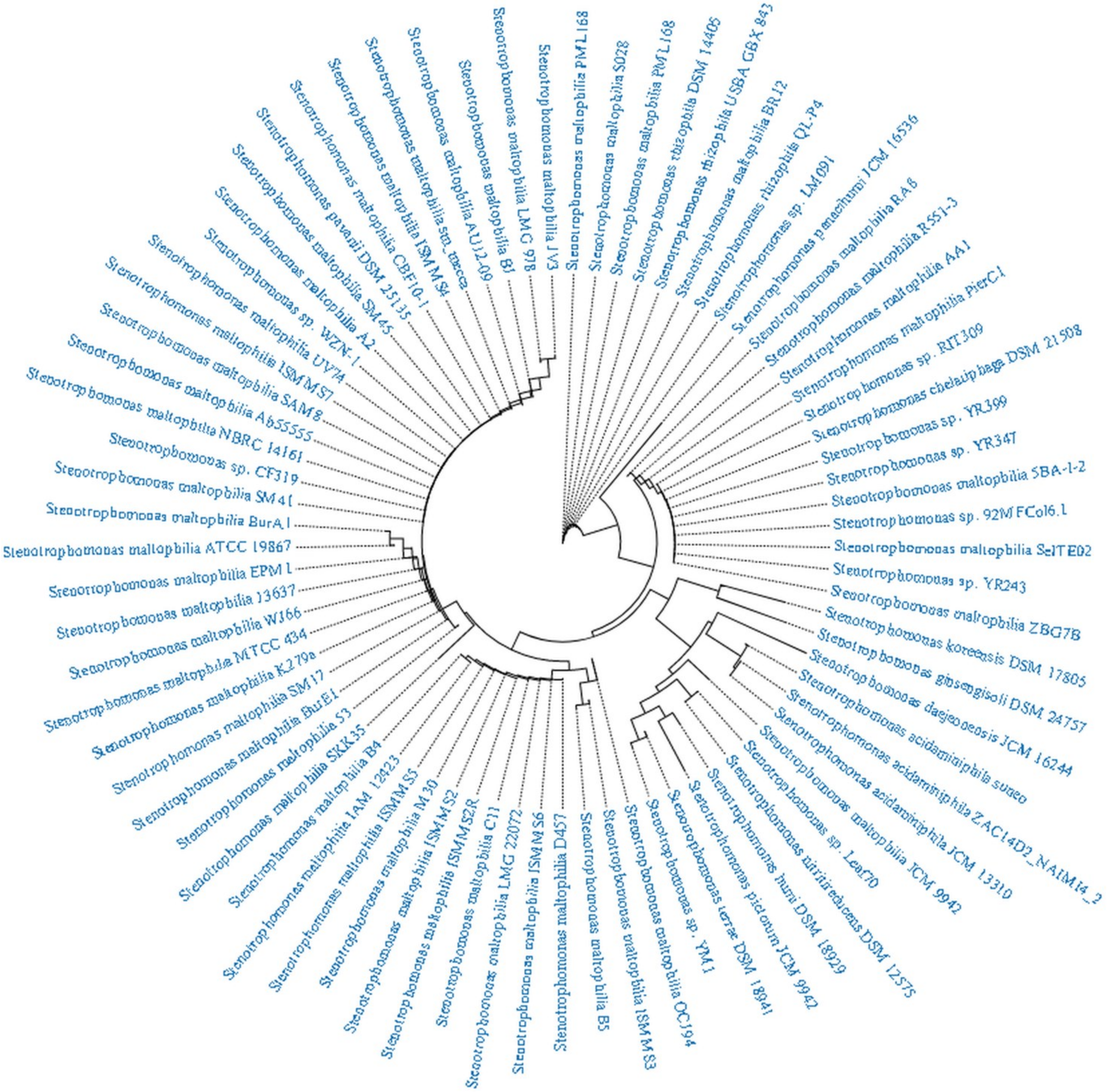
Stenotrophomonas maltophilia PML168
Stenotrophomonas maltophilia S028
Stenotrophomonas maltophilia PML168
Stenotrophomonas rhizophila DSM 14405
Stenotrophomonas rhizophila USBA GBX 843
Stenotrophomonas maltophilia BR12
Stenotrophomonas rhizophila QL-P4
Stenotrophomonas sp. LM091
Stenotrophomonas panacihumi JCM 16536
Stenotrophomonas maltophilia RA8
Stenotrophomonas maltophilia R551-3
Stenotrophomonas maltophilia AA1
Stenotrophomonas maltophilia PierC1
Stenotrophomonas sp. RIT309
Stenotrophomonas chelatiphaga DSM 21508
Stenotrophomonas sp. YR399
Stenotrophomonas sp. YR347
Stenotrophomonas maltophilia 5BA-I-2
Stenotrophomonas sp. 92MFCol6.1
Stenotrophomonas maltophilia SeITE02
Stenotrophomonas sp. YR243
Stenotrophomonas maltophilia ZBG7B
Stenotrophomonas koreensis DSM 17805
Stenotrophomonas ginsengisoli DSM 24757
Stenotrophomonas daejeonensis JCM 16244
Stenotrophomonas acidaminiphila SUNEO
Stenotrophomonas acidaminiphila ZAC14D2_NAIMI4_2
Stenotrophomonas acidaminiphila JCM 13310
Stenotrophomonas maltophilia JCM 9942
Stenotrophomonas sp. Leaf70
Stenotrophomonas nitritireducens DSM 12575
Stenotrophomonas humi DSM 18929
Stenotrophomonas pictorum JCM 9942
Stenotrophomonas terrae DSM 18941
Stenotrophomonas sp. YM1
Stenotrophomonas maltophilia OC194
Stenotrophomonas maltophilia ISMMS3
Stenotrophomonas maltophilia B5
Stenotrophomonas maltophilia D457
Stenotrophomonas maltophilia ISMMS6
Stenotrophomonas maltophilia LMG 22072
Stenotrophomonas maltophilia C11
Stenotrophomonas maltophilia ISMMS2R
Stenotrophomonas maltophilia ISMMS2
Stenotrophomonas maltophilia M30
Stenotrophomonas maltophilia ISMMS5
Stenotrophomonas maltophilia IAM 12423
Stenotrophomonas maltophilia B4
Stenotrophomonas maltophilia SKK35
Stenotrophomonas maltophilia S3
Stenotrophomonas maltophilia BurE1
Stenotrophomonas maltophilia SM177
Stenotrophomonas maltophilia K279a
Stenotrophomonas maltophilia MTCC 434
Stenotrophomonas maltophilia WJ66
Stenotrophomonas maltophilia 13637
Stenotrophomonas maltophilia EPM1
Stenotrophomonas maltophilia ATCC 19867
Stenotrophomonas maltophilia BurA1
Stenotrophomonas maltophilia SM41
Stenotrophomonas sp. CF319
Stenotrophomonas maltophilia NBRC 14161
Stenotrophomonas maltophilia Ab55555
Stenotrophomonas maltophilia SAM8
Stenotrophomonas maltophilia ISMMS7
Stenotrophomonas maltophilia UV74
Stenotrophomonas sp. WZN-1
Stenotrophomonas maltophilia A2
Stenotrophomonas maltophilia SM45
Stenotrophomonas pavanii DSM 25135
Stenotrophomonas maltophilia CBF10-1
Stenotrophomonas maltophilia ISMMS4
Stenotrophomonas maltophilia
Stenotrophomonas maltophilia AU12-09
Stenotrophomonas maltophilia B1
Stenotrophomonas maltophilia LMG 978
Stenotrophomonas maltophilia JV3
