# Supplementary material for: Genome Sequencing and Comparative Analysis of Stenotrophomonas acidaminiphila Reveal Evolutionary Insights Into Sulfamethoxazole Resistance
Source: Front Microbiol. 2018 May 17;9:1013. doi: 10.3389/fmicb.2018.01013 (PMC5966563; doi:10.3389/fmicb.2018.01013)

Supplementary Material

Genome sequencing and comparisive analysis of Stenotrophomonas acidaminiphila reveal evolutionary insights into sulfamethoxazole resistance

Yao-Ting Huang, Jia-Min Chen, Bing-Ching Ho, Zong-Yen Wu, Rita C. Kuo, Po-Yu Liu

*** Correspondence:** Po-Yu Liu: pyliu@vghtc.gov.tw

Supplementary Figures

Supplementary Figure 3. Phylogenetic tree of *S. acidaminiphila* SUNEO and other members of *Stenotrophomonas* species used in this study based on the multilocus sequence analysis of six housekeeping genes (*atpD*, *guaA*, *mutM*, *nuoD*, *ppsA* and *recA*).

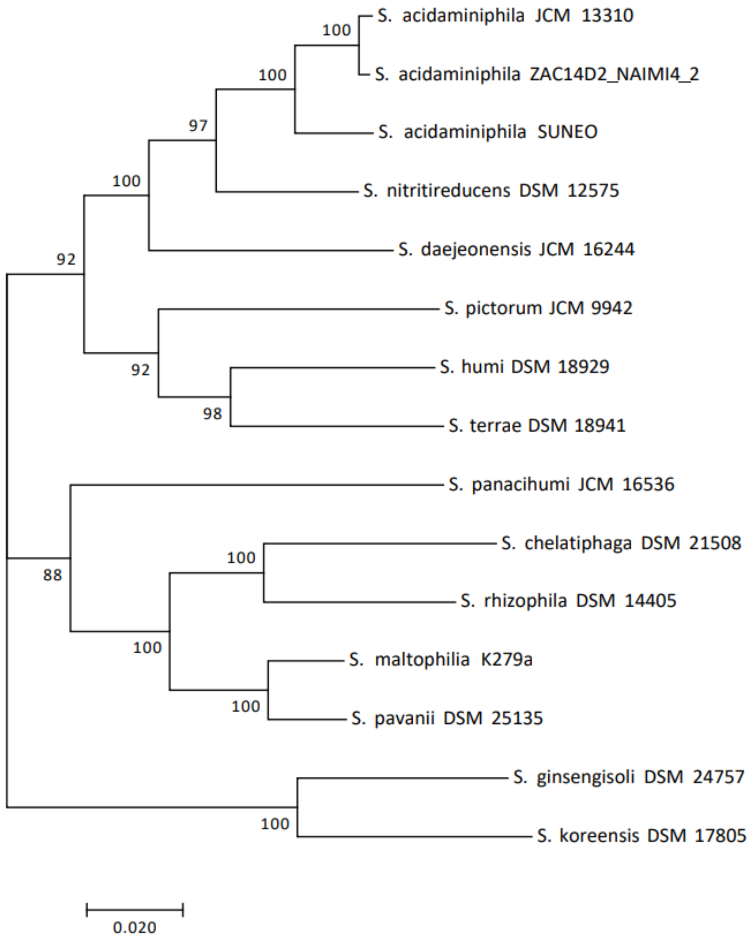

Supplement: Supplementary file 7 [file Image_3.PDF]
